# Small-scale fisheries contribution to food and nutrition security—a case study from Norway

**DOI:** 10.1038/s44183-022-00005-3

**Published:** 2022-10-05

**Authors:** Marian Kjellevold, Grethe Aa. Kuhnle, Svein A. Iversen, Maria W. Markhus, Maria del Mar Mancha-Cisneros, Giulia Gorelli, Kjell Nedreaas

**Affiliations:** 1 Institute of Marine Research, Bergen, Norway; 2 Nicholas School of the Environment, Duke University Marine Laboratory, Beaufort, USA; 3 Center for Marine Conservation and Biodiversity, Scripps Institution of Oceanography, University of California San Diego, San Diego, USA; 4 Fisheries and Aquaculture Division, Food and Agriculture Organization of the United Nations, Rome, Italy

**Keywords:** Lipids, Metals, Proteins, Environmental monitoring, Environmental studies, Agriculture

## Abstract

The Nordic food systems have not been able to reduce the negative development of non-communicable nutrition-related diseases. A shift from a terrestrial animal-based diet toward aquatic foods may enhance the quality of the overall diet and at the same time contribute to climate change mitigation. The aim of the present study is to quantify catches from the Norwegian small-scale fisheries (SSF), compare the catches to recommended dietary intakes, and assess the potential contribution of SSF to local food and nutrition security (FNS). The Norwegian SSF catches are landed in Norway, and thus highly accessible in times of crisis. Here we show that the Norwegian SSF can provide a population of 5 million people with 1–2 portions of seafood weekly (360 g), corresponding to ~70–96% of the recommended intake/person/year of the key nutrients such as vitamin B12, iodine, and the n-3 long-chained poly-unsaturated fatty acid docosahexaenoic acid. These findings provide a basis for policymakers on the potential of the SSF to substantially contribute to national FNS. We call for a more offensive policy where fish as a source of essential micronutrients are implemented in food-based dietary guidelines as an alternative to supplement and fortify other foods.

## Introduction

Unhealthy diets are a leading risk factor for poor health. The Nordic food systems have not been able to reduce the negative development of non-communicable nutrition-related diseases^1^, and micronutrient deficiency, e.g. vitamin D^2,3^, vitamin B12^4^, selenium^5^, and iodine^6,7^, are prevalent in some age groups and life stages. Aquatic foods play an important role in the global food provision, accounting for about 17% of animal protein, and 7% of all protein consumed globally^8^. Fish as a food group is also a rich source of bioavailable micronutrients, and nutrients in marine catches may contribute to the dietary requirements of several micronutrients^9,10^. In Norway, fish is a major dietary source of vitamin D and vitamin B12^11^ and the food commodity with the highest nutrient content of iodine and marine n-3 fatty acids. Yet, the potential contribution of fish to food and nutrition security (FNS) is all but ignored in both the national and international debate^12^, and under-researched compared to the other food groups in FNS^13^. A shift from terrestrial animal-based diets toward aquatic foods may enhance the overall diet and at the same time contribute to climate change mitigation^14^.

Norway benefits from a safe and sufficient food supply but the overall self-sufficiency (energy requirements for the population) was only 45% in 2020 of which 80% from fish^15^. Nationally produced food and coverage would in a crisis be higher (89%) but would foresee increased intake of plant-based foods and reduction in export of fish. Norwegian coverage of fish is 20-fold the Norwegian population energy requirements when taking export into account. In 2019, 2.4 million tons of fish were exported, while 207,000 tons were imported^15^. In the US, the COVID-19 crisis in 2020 resulted in substantial declines in fresh seafood catches (40%), imports (37%), and exports (43%) relative to the previous year, while frozen seafood products were generally less affected^16^. In the North-Eastern Adriatic Sea, fishing activity was reduced by 84% during lockdown but resumed in the third week of April 2020^17^. However, in Norway, the export of seafood (including farmed salmon) decreased by only 2% in 2020 compared to 2019^18^.

Fisheries are a diverse sector using a variety of fishing techniques and gears to harvest wild aquatic resources. The sector is divided into small-scale fisheries (SSF) and large-scale fisheries (LSF). Both classification and terminology regarding (SSF) vary from country to country, and neither the Food and Agriculture Organization of the United Nations (FAO) nor the European Union (EU) has harmonized or found it possible or useful to formulate a universal definition. Even though SSFs often are recognized as having low productivity and yield rates, modern SSFs can be economical efficient^19^.

Describing the nutritional contribution of small-scale fisheries (SSF) will add valuable data to better understand the role of these fisheries to national FNS. Thus, the aim of this study is to quantify catches from both the Norwegian SSF, compare these catches to recommended dietary intakes, and assess the potential contribution of these fisheries to local FNS.

## Material and methods

The present study is based on the Norwegian country case study of the Illuminating Hidden Harvests (IHH) project, a collaborative study led by FAO, Duke University, and WorldFish to assess and quantify the global contributions and impacts of small-scale fisheries to the economic, social, and environmental dimensions of sustainable development, with additional emphasis on FNS and governance issues around SSF. Overall, the IHH study aims to better inform policy-making processes and empower fishing communities with key information about the contributions of SSF to sustainable development goals. Within IHH, 58 country case studies were conducted around the globe (including Norway) to produce national-level estimates of key indicators disaggregated for SSF and within a multi-dimensional framework to highlight the links between different contributions of SSF, including the one between SSF production, local FNS, and food safety. Through comprehensive work have extracted data from official Norwegian statistical- and other relevant sources to adapt the data to fit into the IHH database^20^. The Norwegian data are stored in the IHH database and in the Institute of Marine Research’s database.

### Fisheries in Norway

The Norwegian SSF fleet is in this study defined as commercial vessels less than 15 m fishing inside 12 nautical miles and in the fjords^20^. The number of vessels defined as part of this fleet was ~25,000 in 1980 and has decreased to 5004 in 2017. The average age of the vessels in 2017 was about 23 years and the average crew on board the smallest vessels (<11 m) was 1.6 persons and on the larger vessels (11–14.99 m) 2.7 persons. The same vessels from this fleet take part in different fisheries during the year in different areas and with different gears.

LSF is defined as all vessels above 15-m, fishing both outside and inside of 12 nautical miles. The Norwegian LSF fleet is mostly active in the International Council for the Exploration of the Sea (ICES), Northwest Atlantic Fisheries Organization (NAFO), and to some extent in the Commission for the Conservation of Antarctic Marine Living Resources (CCAMLR) areas, and occasionally in joint ventures in other areas.

The Norwegian catches by the SSF and LSF are well accounted for in the Fisheries statistics by the Norwegian Directorate of Fisheries and thereby not hidden harvest. However, the statistics do not account for the recreational fishery, subsistence fishery, and that the fishermen are allowed to bring home 100 kg of fish for home cooking. We have estimated that the fishermen bring home about 1000 tons per year. The not traded part of the Norwegian subsistence fishery, i.e. the real subsistence fishery, has in this project been set to 46,000 tons per year, hereof ~23,000 tons cod, 8500 tons saithe, 8500 tons mackerel, and 6000 tons “other fish” species^20^. The present study does not include the inland fishery that is managed by a different ministry and directorate. However, those catches are negligible. There are no official catch statistics from this fishery, but catches are estimated at 8000–10,000 tons per year^21^. Recreational fisheries are not included in this study.

### Data sources

We have retrieved and adapted data available from the Norwegian Directorate of Fisheries, the Institute of Marine Research, and data directly available on the websites of relevant institutions. A detailed description can be found in Nedreaas et al.^20^.

The analytical data are retrieved from the open access food composition database Seafood data^22^ and detailed information on the year analyzed and a number of samples (*n*) are given in Supplementary material, Tables 1–11. For iodine, values for cod, saithe, and haddock were retrieved from Nerhus et al. (2018)^23^. All chemical analyses are performed at the laboratories at the Institute of Marine Research, Norway. The laboratory participates in national and international proficiency tests with satisfactory results to assess the accuracy and precision of the analyses, in addition to the measurement uncertainty of each method. The analyses were performed using accredited methods according to ISO 17025:2005, and Certified Reference Materials (CRM) were included in each sample run. Overview of methods, Limits of Quantification (LoQ) and measurements of uncertainties are described in detail in Reksten et al. (2020)^24^.

Data on seafood intake was retrieved from NORKOST 3, a national dietary survey conducted among adults in Norway. During 2010–2011, the diet was assessed in a population of 862 men and 925 women between 18 and 70 years of age using two randomly distributed 24-h recalls^11^. Previously published data do not include detailed information on seafood consumption. Thus, new de-aggregated data on the consumption of seafood at the species level have been retrieved by the Institute of Marine Research, especially for this publication. The data were weighted for educational level as the data collected in Norkost3 were skewed in terms of education. For e.g. male participants, education levels were 50% with low and 50% with a high level of education, while men in the general population were 74% with low and 26% with a high level of education. When weighing, participants with low education were given more weight by gaining a factor attached to it; the share of low education in the population divided by the share with low education in the sample material. The participants with a high level of education were given a lower weight according to the same method.

### Calculations

Tons from edible parts (fillet) from SSF were calculated by dividing the average live-weight catch (2013–2017) by the conversion factor for each species (Table 1). The category “other fish” consist of several different species which unfortunately have no conversion factors available. Thus, we applied an average value for the mixed (other) fish species. The total amount of each nutrient (I, Fe, Zn, Ca, Vit A, Vit D, Vit B12, and the very long-chain polyunsaturated n-3 fatty acid (n-3 LCPUFA) docosahexaenoic acid (DHA)) was calculated by multiplying the estimated tons of fillet for each species provided from SSF (Table 1) with the nutrient content of each species (Supplementary material, Tables 1–9). For haddock and edible crab, no analytical data was available for Vit A, and for haddock values for Vit D, Vit B12, and DHA were also missing. Missing values were therefore retrieved from the Norwegian Food Composition table^25^. For the category “other fish”, a conservative approach was chosen, and the nutrient value was set to the lowest analytical value of the five species with analytical data. The total population of Norway was set to 5 million, and recommended intake (RI) for each nutrient was retrieved from the Nordic Nutrition Recommendations for women aged 18–60 (NNR, 2012). A number of RI portions/person/year potential provided by SSF (Table 2) were calculated by using estimated tons of filet of each species (Table 1), divided with portion size (RI for the given nutrient divided with the nutrient content given in Supplementary material, Tables 1–9), divided with an estimated population of 5 million.

**Table 1:** Average small-scale fisheries (SSF) catch (2013–2017) and average first-hand values (NOK) for the most important species groups in the SSF fishery and their relative importance within the total Norwegian fishery.

Species		Traded				Not-traded			Total (traded + non-traded)	
Common name	Latin name	Tons live weight	Mill NOK	Catch^a^	Value^a^	“Taken home” ^b^	Subsistence Fisheries^c^	Conversion factor, Fillet^d^	Estimated tons, live weight	Estimated tons, fillet SSF
Cod	*Gadus morhua*	129,552	1395	5.8	8.6	500	23,000	3.43	153,100	45,000
Herring	*Clupea harengus*	22,758	115	1.0	0.7	ND	ND	2.44	22,800	9000
Saithe	*Pollachius virens*	21,142	156	0.9	1.0	200	8500	3.77^e^	29,800	8000
Haddock	*Melanogrammus aeglefinus*	16,116	143	0.7	0.9	ND	ND	3.28	16,100	5000
Mackerel	*Scomber scombrus*	12,286	82	0.5	0.5	200	8500	2.60	21,000	8000
Other fish^f^		20,826	530	0.9	3.3	100	6000	3.1^g^	26,900	8700
Edible crab	*Cancer pagurus*	4799	52	0.2	0.3	ND	ND	4.0^h^	4800	1200
Shrimp	*Pandalus borealis*	3592	174	0.2	1.1	ND	ND	3.3^i^	3600	1100
King crab	*Paralithodes camtschaticus*	1901	198	0.1	1.2	ND	ND	4.0^h^	1900	500
Other evertebrates^j^		420	35	0.0	0.2	ND	ND	ND^k^	400	ND
Total		233,392	2880	10.4	17.7					86,500

ND—no data.

^a^SSF % of total Norwegian fishery (small-scale fisheries + large-scale fisheries).

^b^Taken home by the fishermen (non-traded).

^c^Subsistence fisheries (non-traded). Data retrieved from Hallenstvedt and Wulff (2004)^45^.

^d^Statistics Norway (2020)^18^.

^e^96% of SFF catch of Saithe is north of 62N, conversion factor 3.80, 4% is south of 62N, conversion factor 3.00. The weighted mean is set to 3.77.

^f^This group contains 66 species including sharks and skates.

^g^No conversion factor available, used the mean of the five species with conversion factor.

^h^No conversion factor available, used edible part (33%) as defined in the Norwegian Food Composition Table^25^.

^i^No conversion factor available, used edible part (40%) as defined in the Norwegian Food Composition Table^25^.

^j^Clams, snails, crustaceans.

^k^No conversion factor available.

**Table 2:** Number of daily recommended intakes (RI)/year/person provided by small-scale fisheries (SSF) catches given a total population of 5 million people and RI for adult women.

Species, common name	Species, Latin name	Estimated g/week/person	Vitamin A1 (retinol)	Vitamin D3	Vitamin B12	Iron	Iodine	Zinc	DHA
RI			700 ARE	10 µg	2 µg	15 mg	150 µg	7 mg	250 mg
Cod	*Gadus morhua*	173	1	0	45	1	114	5	76
Herring	*Clupea harengus*	35	1	50	112	1	2	2	50
Saithe	*Pollachius virens*	31	0	0	27	0	84	1	20
Haddock	*Melanogrammus aeglefinus*	19	0^a^	1^a^	66^a^	0	27	0	2^a^
Mackerel	*Scomber scombrus*	31	0	6	80	1	2	1	130
Other fish^b^		32	2	0	9	0	2	1	15
Edible crab	*Cancer pagurus*	14	0^a^	0	6	0	18	3	0
Shrimp	*Pandalus borealis*	18	0	0	4	0	5	0	1
King crab	*Paralithodes camtschaticus*	7	0	0	3	0	4	1	0
Total RI portions^c^			4	57	352	3	258	14	294

Recommended intake (RI) for women 18–60 years old (NNR, 2012). Retinol equivalents (RE); 1 retinol equivalent = 1 µg retinol.

*DHA* docosahexaenoic acid, an omega-3 fatty acid.

^a^No analytical data available. Nutrient values retrieved from the Norwegian Food Composition Table^25^.

^b^This group contain 66 species including shark and skates. Nutrient values set to the lowest analytical value of the five fish species; Vitamin A1 (cod), vitamin D3 (<Limit of Quantification), vitamin B12 (cod), iron (cod), Iodine (herring), zinc (haddock), and DHA (cod).

^c^Clams, snails, and crustaceans are not included due to no conversion factor.

## Results

### Catch

In the official Norwegian statistical sources landings and not catches are reported. Catches are occasionally treated before landing to eliminate undersized fish or protected species, or the catch is high-grade to increase the economic value. In the present paper, catch means landing, and Table 1 shows the average catch (2013-2017) and average values for the most important species groups in the SSF fishery and their relative importance within the total Norwegian fishery (SSF + LSF) are given. The LSF fishery is the dominant part of the Norwegian fishery both in terms of volume and value.

The total yearly SSF catch has been quite stable at about 222,000–256,000 tons for 2013–2017 with an average of 233,392 tons which is 10.4% of the average total Norwegian catch (SSF + LSF), and 8.6% of the total value of this catch (Table 1). The Norwegian SSF fisheries exploit more than 100 different species, of which about 70 different species are commercial. Cod dominates the SSF fishery accounting for 5.8% of volume and 8.8% of the value of the total Norwegian fishery. The SSF fleet catches on average 29.5% of the total Norwegian catch of cod. This fishery exploits two different oceanic cod stocks, i.e. North East Arctic cod and North Sea cod, and three coastal cod stocks or management units. The coastal cod is on a low level and the fleet is dependent on sound coastal cod populations which currently, however, are under rebuilding. It is hence expected that the temporary burden carried by the SSF to rebuild the coastal cod will result in higher sustainable cod catches for the SSF in the future. Firsthand value of the total SSF catch increased from 2064 mill NOK in 2013 to 3562 mill NOK in 2017 (currently, 100 NOK = 10.5 EUR/8.9 USD), with an average value of 2880 mill NOK which is 17.7% of the value of the total Norwegian fishery. Cod and herring are the most important species both in tonnage and value of the Norwegian SSF and LSF fisheries. The same species can belong to different stocks/management units that are managed individually by the Norwegian Directorate of Fisheries/Ministry of Fisheries. Most of the stocks caught by the Norwegian SSF are evaluated by ICES. Of the 2017 SSF catch, 93.4% was from stocks evaluated as sustainable, 0.6% not sustainable and 6% from stocks not evaluated. About 90% of the Norwegian SSF are regulated by quotas and the rest by other regulations like minimum/maximum legal size, open/closed fishing areas or seasons, by-catch regulations, and/or discard bans by Norwegian authorities.

### Nutrient content and potential contribution to FNS

Analytical values for the selected nutrients in fillets for the eight most important species in SSF are shown in Supplementary material, Tables 1–8. The total fat content varies from 1–18 g/100 g and is highest in herring and mackerel. These two species are also the richest sources of fatty acid DHA and the only species with vitamin D3 values above the LoQ. All species, however, (except haddock where no analytical data were available for these nutrients) will cover the daily RI for DHA and vitamin B12. The lean fish species saithe, haddock and cod are the best sources of iodine, and one portion (270 g)^26^ will cover the RI for adult women 3–14-times. Cod liver is a rich source of fat (55 g/100 g, *n* = 90, 2019), vitamin A (486 µg/100 g, *n* = 27, 2006), vitamin D (9.4 µg/100 g, *n* = 26, 2006) and DHA (5710 mg/100 g, *n* = 31, 2006)^22^.

The fish catch from SSF contributes approximately 70%, 80%, and 96% of the RI/year/person for iodine, DHA, and vitamin B12, respectively, for a population of 5 million (Table 2). Even though haddock has the highest content of iodine and mackerel and herring has the highest DHA values of the species included (Supplementary material, Tables 1–9), cod is the most important source of iodine and DHA due to the high volumes (Table 1). Herring is the species with the highest vitamin B12 content and is the most important B12 source. The SSF catches are not good sources of vitamin A1, iron, and zinc when only considering the fillet from these species as food. A portion of 200 grams of herring and mackerel will cover the daily RI of vitamin D, vitamin B12, and DHA, while saithe, haddock, and cod will cover the daily RI of iodine. A portion of cod liver (80 g) will cover 56% and 75% of the RI for vitamins A and D, respectively.

The mean seafood intake of adults 18–70 years old is 469 g/week (67 g/day) and median seafood intake is 210 g/week (30 g/day) among all participants (*n* = 1787), respectively (Table 3). The data is skewed, 35% report zero seafood intake, and 25% report a daily intake corresponding to more than 700 g of seafood weekly (109 g/day). The mean seafood intake among those reporting seafood intakes above zero (*n* = 1158) is 728 g/week (104 g/day), and the median intake 581 g (83 g/day) (Table 4), meaning that >50% of the participants reporting a seafood intake reported an intake above the recommended 300-450 g fish weekly^27^. When considering only those reporting seafood intakes above zero during the survey (*n* = 1158), a more diverse pattern is, however, observed with <10 respondents for most of the species/food items reported (Supplementary material, Table 12).

**Table 3:** Seafood intake (g/day) among adults 18–70 years old in Norway (*n* = 1787)^a^ (mean g/day (SD), 25-, 50- (median), 75-, 95-percentiles of edible parts^b^).

	*n*	Mean (SD)	Min	Max	Percentiles				
					5	25	50	75	95
Total Seafood	1787	67 (88)	0	826	0	0	30	109	237
Fatty fish^c^	1787	15 (38)	0	418	0	0	0	0	99
Lean fish^d^	1787	16 (51)	0	714	0	0	0	0	59
Unspecified fish^e^	1787	6 (26)	0	250	0	0	0	0	37
Fish products^f^	1787	13 (40)	0	481	0	0	0	0	93
Fish breadspread^g^	1787	10 (25)	0	191	0	0	0	9	56
Shellfish	1787	4 (17)	0	250	0	0	0	0	29
Fish liver/roe	1787	1 (8)	0	139	0	0	0	0	0
Fish dishes^h^	1787	3 (24)	0	423	0	0	0	0	0

^a^Norkost 3 is the third national dietary survey conducted among adults in Norway. During 2010–2011, the diet was assessed in a population of 862 men and 925 women (1787 participants in total) between 18 and 70 years of age. The method used was two randomly distributed 24-h recalls.

^b^Note that 1158 participants in total reported a seafood intake above zero, but the mean is based on the number of participants reporting for each category and thus does not necessarily add up to the total number of participants.

^c^Total fat >5%, salmon, trout, halibut, mackerel, and herring, in descending order.

^d^Total fat <5%, cod, saithe, haddock, and other in descending order.

^e^Assorted fish from dishes (wok, salad, etc.).

^f^Processed <100% fish products (burger, pudding, breaded, fried, etc.).

^g^Mackerel in sauce, canned tuna, herring in sauce, caviar, anchovies, sprat, and pate, in descending order.

^h^Soup, sushi, and fish au gratin.

**Table 4:** Seafood intake (g/day) by adults 18–70 years old among only those reporting a seafood intake above zero^a^ (mean g/day (SD), 25-, 50- (median), 75-, 95-percentiles of edible parts^b^).

	*n*	Mean (SD)	Min	Max	Percentiles				
					5	25	50	75	95
Total Seafood	1158	104 (90)	2	826	10	36	83	145	276
Fatty fish^c^	359	73 (55)	8	418	15	30	60	99	166
Lean fish^d^	232	120 (84)	13	714	36	63	105	156	265
Unspecified fish^e^	106	92 (54)	1	250	13	57	82	118	217
Fish products^f^	265	90 (62)	7	481	25	55	75	117	181
Fish breadspread^g^	564	33 (35)	3	191	4	10	20	43	103
Shellfish	217	36 (35)	1	250	5	13	25	50	100
Fish liver/roe	15	71 (48)	2	139	8	21	80	106	139
Fish dishes^h^	35	153 (80)	33	423	77	113	129	170	322

^a^Norkost 3 is the third national dietary survey conducted among adults in Norway. During 2010–2011, the diet was assessed in a population of 862 men and 925 women (1787 participants in total) between 18 and 70 years of age. The method used was two randomly distributed 24-h recalls.

^b^Note that 1158 participants in total reported a seafood intake above zero, but the mean is based on the number of participants reporting for each category and thus does not necessarily add up to the total number of participants

^c^Total fat >5%, salmon, trout, halibut, mackerel, and herring, in descending order

^d^Total fat <5%, cod, saithe, haddock, and other in descending order

^e^Assorted fish from dishes (wok, salad, etc.),

^f^Processed <100% fish products (burger, pudding, breaded, fried, etc.)

^g^Mackerel in sauce, canned tuna, herring in sauce, caviar, anchovies, sprat, and pate, in descending order.

^h^Soup, sushi, and fish au gratin.

### Food safety

Data on total mercury and dioxin and dl-PCBs are given in Supplementary material, Tables 10 and 11, respectively. The mean concentration of dl-PCBs in cod liver is 16.4 nanogram TEQ/kg (*n* = 67, 2019) ranging from 2.73 to 93.1 ng TEQ/kg (median 6.19 ng TEQ/kg). Dividing the total estimated catch of fillet for each species available from SFF on a population of 5 million people, give a weekly intake of 173 g cod, 31 g of saithe, 19 g of haddock, and 35 g herring, and 31 g of mackerel. Thus, an estimated total weekly intake of 288 g/person/week, and approximately 1/3 from oily fish (mackerel and herring), is available from the SFF catch which is within the recommended intake of 300–450 g per week^27^. If the “other fish” is added, the intake is 320 g, and when adding weekly portions of crustaceans and invertebrates, the total weekly intake per person adds up to 360 g/person/week which is well within the recommended intake (Table 2).

## Discussion

The fish resources harvested by the SSF are of high importance for FNS in Norway. The resources are harvested within 12 nautical miles of the Norwegian economic zone and are landed locally, thus being a highly accessible food source in case of crisis. Even though the COVID-19 situation did not have a major impact on access to food, Norway has experienced times of food shortage during and between the two world wars. Studies from 1912 to 1913 and until 1937 showed that fish contributed between 1.7% and 5.9% of the total energy intake, but potentially could cover 20% of the energy demands^28^. This is in accordance with the present findings as all catch presently available in the SSF could potentially have covered 20% of the energy intake of the Norwegian population if consumed locally. If taking both LSF and SSF into consideration, fish can cover energy demand 20-fold the Norwegian population^15^. Evang (1939) emphasised that larger quanta of fish would not be feasible to consume unless new products were developed. Semi-industrialized dishes made from sustainable fish species can be valuable options for meeting nutritional needs of target populations^29^. Randomized intervention studies with fish have shown that it is possible to increase fish intake of both lean and oily fish species when high-quality products and dishes are made available^30–32^. Thus, development of semi-industrial dishes, e.g. kindergartens and institutions could increase the access and consumption of fish.

The Norwegian SSF has the potential to provide a population of 5 million people with 1–2 portion of seafood (360 g) weekly corresponding to approximately 70–96% of the population RI demands/year for the key nutrients vitamin B12, iodine, and the n-3 LCPUFA DHA (Table 2). The most important fish species in the SSF are cod, herring, saithe, haddock, and mackerel. The two oily species, mackerel, and herring are superior when it comes to the content of DHA due to the high-fat content (Supplementary material, Tables 2 and 9), but cod contribute with comparable RI portions due to higher catch volume (Table 1). For iodine, saithe are the species, which is superior in content, but due to volume, cod contribute with most RI portions. None of the fish species (fillet samples) were rich sources of vitamin A, iron, or zinc, and for vitamin D, herring is the only relevant species contributing with 50 RI portions/person/year. It is important to keep in mind, that there can be great individual variation in content between individual fish of a species. The greatest variation was found for vitamin B12, iodine, and DHA (Supplementary material Tables 4, 7, and 9). High variation in content is a challenge for food composition compilers as usually only one value is reported in food composition tables. We have previously shown that iodine content in Atlantic cod varies between 22 and 720 μg/100 g, saithe from 35 to 820 and haddock from 35 to 2200 μg/100 g^23^. Thus, more data on variables that can explain variation are warranted.

In the present study, we have not taken by-products into consideration. The SSF provides a total of 311 million meals adding to 90,000 tons which means that 190,000 tons have the potential to be used in other by-products such as liver oil, fish meal, animal food, or silage. According to data available from the Directorate of Fisheries, a grand total of 4.4 tons liver and 3.5 tons roe were landed in 2017. The mean intake of fish liver and roe in NORKOST 3 is 1 g/day for all participants (Table 3) and 71 g/day for those participants reporting seafood intake above zero during the study (Table 4). One portion of cod liver (80 g) will contribute more than 50% of RI for vitamins A and D but will also be a source of dioxins and dl-PCBs. The European Food Safety Authorities (EFSA) has assessed the risk for human health related to the presence of dioxins and dioxin-like PCBs in food and the tolerable weekly intake (TWI) was recently reduced from 14 to 2 pg WHO TEQ-05/kg body weight^33^. The Norwegian Scientific Committee for Food and Environment (VKM) recently published an updated risk-benefit assessment of fish in the Norwegian diet concluding that the benefits of increasing fish intake to the recommended two to three dinner courses per week (corresponding to 300–450 g, including at least 200 g fatty fish in adults) outweigh the risks for all age groups^34^.

Shrimp and crab are lean foods with similar nutritional qualities as lean fish except for being a richer source of zinc (Supplementary material, Table 8). According to the most recent representative dietary survey among adults, fish and fish products contribute 40% of vitamin D, 34% of vitamin B12, 2% of iron, and vitamin A^11^. Farmed salmon is the most consumed oily fish and cod is the most consumed lean fish, however, the intake is highly skewed (Supplementary material Table 12).

The IHH approach and its focus on the multi-dimensional nature of SSF suggests a framework that allows the use of multiple data sources and methodologies to connect important aspects of SSF beyond production levels, which can further increase the impact on the role of SSF to FNS The strength of this study lies in the connection between compiling existing SSF data and nutrient content to address FNS. The relevance of this study is strengthened by having high-quality analytical data on the nutritional value and the potential hazards (contaminants), considering both risk and benefits when assessing the role of SSF for FNS. We have, however, identified data gaps; no conversion factor from live weight to edible part for shrimps and crabs is available, analytical data were missing for key nutrients in haddock and the sample size for some nutrients is relatively low (*n* = <10). As shrimps and crabs are important foods in times of crisis, more data on nutrient composition and conversion factors are warranted. A strength is that we have included detailed data on fish consumption from NORKOST3 not previously published. However, NORKOST3 is based on two 24-h recalls that may not capture the intake of the highly diverse group of seafood. Thus, specific dietary surveys on seafood consumption are needed.

Internationally, the reference diet suggested by the EAT-Lancet Commission on healthy diets from sustainable food systems has received much attention^35^. The proposed intake of fish and shellfish is 28 g/day (range 0–100 g), corresponding to a weekly intake of 196 g (range 0–700 g). This is close to the median intake in the Norwegian population (NORKOST3), but approximately half the present Norwegian recommendation of 300–450 g of seafood per week. In a global context, daily consumption of 28 g would lead to a need for a substantial increase in seafood production^36^. However, for Norway, no increase in catches is needed, and an increase in consumption to reach the national recommendations can be covered by SFF alone. This is of utmost importance to the Norwegian population as wild-caught fish is a recommended and highly nutritious food source that can be sustainably harvested all year round. Only 3% of the total land area in Norway is cultivated and will not be able to provide its population self-sufficiently with a plant-based diet^37^. Food security exists only when all people at all times have access to sufficient, safe, and nutritious food. In this study, we have shown that the Norwegian SSF has the potential to fulfill the six dimensions of food security. SSF supplies the population with the recommended intake of safe and nutritious seafood (“agency”, “availability” and “utilization”). However, even though the catch from SFF is sustainable, available, and stable (“stability” and “sustainability”), the intake is lower than what is recommended (“access” and “agency”). There is a potential to use even more of the SSF catch directly as food since some of this catch (roughly 5000 tons per year) is not landed due to price, low season, quota exhaustion, damaged fish, etc., but still relevant for human consumption^38^. There are knowledge gaps and data scarcity on seafood consumption, and no data available on the source of seafood (relative consumption from the different fisheries; SSF, LSF, recreational fisheries). There is a need for dietary surveys using an optimal methodology for assessing the intake of seafood, and nutrient data on haddock and shellfish. A shift from terrestrial animal-based diets towards aquatic foods may enhance the overall diet^9^ including reducing micronutrient deficiency of iodine^39^ and vitamin B12. Thus, these data document that seafood from SSF can contribute significantly to micronutrients when consumed in accordance with the recommendations. An increase in aquatic food production may also contribute to climate change mitigation^14^. The Nordic and Baltic countries are presently developing a common scientific basis (the Nordic Nutrition Recommendations) for national nutrition recommendations and will have a special emphasis on the integration of aspects related to, e.g. sustainability^40^. Feed production and fuel are factors that have a major impact on the carbon footprint of Norwegian seafood products^41^. The average footprint for the Norwegian SFF, defined as the emission of CO_2_ was only 1/20 of the LSF footprint during 2013–2017^20^. Thus, we agree with Meltzer et al. (2019) that “balancing import and export in the Nordic countries, subject to minimization of environmental impact, maybe a basic principle in future Nordic food policy”^42^ SSF vessels that operate close to land and spread along the entire coast will be a reliable and sustainable supplier of nutrients, also during crises, as long as they have energy for transport. Our findings provide a basis for policymakers on the potential of the SSF to substantially contribute to national FNS. To increase the contribution of seafood to FNS, coherent policies among sectors linking fish production to consumers are needed^43^, and the seafood resources must be acknowledged as a biodiverse and nutritious food source. We call for a more offensive policy where fish as a source of essential micronutrients are implemented in the food-based dietary guidelines, as an alternative to supplement and fortify other foods. This is a precaution for reaching the Norwegian National Action Plan for a Healthier Diet on increasing the population's seafood intake by 20%^44^.

### Reporting summary

Further information on research design is available in the Nature Research Reporting Summary linked to this article.

## Supplementary information

Supplementary Checklist

Reporting Summary

Supplementary material Table 12

Supplementary Tables S1–S11

## Data Availability

The data that support the findings of this study are available from the corresponding author upon reasonable request.

## References

[1] Wood, A. et al. *Nordic Food Systems for Improved Health and Sustainability: Baseline Assessment to Inform Transformation* (Swedish Stockholm Resilience Center, Stockholm University, 2019).

[2] Oberg, J., Jorde, R., Almas, B., Emaus, N. & Grimnes, G. Vitamin D deficiency and lifestyle risk factors in a Norwegian adolescent population. *Scand. J. Public Health* **42**, 593–602 (2014). 25053469 10.1177/1403494814541593

[3] Larose, T. L. et al. Factors associated with vitamin D deficiency in a Norwegian population: the HUNT Study. *J. Epidemiol. Community Health* **68**, 165–170 (2014). 24197920 10.1136/jech-2013-202587

[4] Green, R. et al. Vitamin B(12) deficiency. *Nat Rev Dis Primers* **3**, 17040 (2017). 28660890 10.1038/nrdp.2017.40

[5] Varsi, K. et al. Impact of maternal selenium status on infant outcome during the first 6 months of life. *Nutrients* **9**, (2017) 10.3390/nu9050486. 10.3390/nu9050486 PMC5452216 28492511

[6] Aakre, I. et al. Iodine status during pregnancy and at 6 weeks, 6, 12 and 18 months post-partum. *Matern. Child Nutr.* **17**, 10 (2021). 10.1111/mcn.13050 PMC7729798 32602197

[7] Brantsaeter, A. L. et al. Inadequate iodine intake in population groups defined by age, life stage and vegetarian dietary practice in a Norwegian convenience sample. *Nutrients* **10**, 10.3390/nu10020230 (2018). 10.3390/nu10020230 PMC5852806 29462974

[8] FAO. *The State of World Fisheries and Aquaculture—Sustainability in Action* (The Food and Agriculture Organization of the United Nations, Rome, 2020).

[9] Golden, C. D. et al. Aquatic foods to nourish nations. *Nature* **598**, 315–+ (2021). 34526720 10.1038/s41586-021-03917-1 PMC10584661

[10] Aakre, I. et al. New data on nutrient composition in large selection of commercially available seafood products and its impact on micronutrient intake. *Food Nutr. Res.* **63**, 12 (2019). 10.29219/fnr.v63.3573 PMC6642616 31360148

[11] Totland, T. et al. *Norkost 3—En landsomfattende kostholdsundersøkelse blant menn og kvinner i Norge i alderen 18–70 år, 2010–11* (Nationwide dietary survey in Norway among men and women aged 18–70 years, 2010–11) (University of Oslo, the Norwegian Food Safety Authority, Norwegian Directorate of Health, Oslo, Norway, 2012).

[12] Bene, C. et al. Feeding 9 billion by 2050—putting fish back on the menu. *Food Secur.* **7**, 261–274 (2015).

[13] Stetkiewicz, S. et al. Seafood in food security: a call for bridging the terrestrial-aquatic divide. *Front. Sustain. Food Syst***. 5** (2022).

[14] Hoeg-Guldberg, O. et al. *The Ocean as a Solution to Climate Change: Five Opportunities for Action* (World Resources Institute, Washington, DC, USA, 2019).

[15] Norwegian Directorate of Health. *Utvikling i norsk kosthold 2020. Matforsyningsstatistikk* (Norwegian Directorate of Health, Helsedirektoratet, Oslo, 2021).

[16] White, E. R. et al. Early effects of COVID-19 on US fisheries and seafood consumption. *Fish. Fish.* **22**, 232–239, 10.1111/faf.12525 (2021). 10.1111/faf.12525 PMC7753393 33362433

[17] Depellegrin, D., Bastianini, M., Fadini, A. & Menegon, S. The effects of COVID-19 induced lockdown measures on maritime settings of a coastal region. *Sci. Total Environ.* **740**, 8 (2020). 10.1016/j.scitotenv.2020.140123 PMC7286833 32554030

[18] Statistics Norway. *Export of Fish, by Country/trade, Region/continent 2007–2020* (Statistics Norway, 2020).

[19] Word Bank. *Hidden Harvest—The Global Contribution of Capture Fisheries* Vol. 92 (Word Bank, Washington, DC, 2012).

[20] Nedreaas, K. H., Kuhnle, G. A., Iversen, S. A. & Kjellevold, M. *The Norwegian Small Scale Fishery and Data Provided for the IHH FAO-Duke-WorldFish Project*. *Rapport fra havforskningen* (Norwegian Directorate for Nature Management, 2022).

[21] Norwegian Directorate for Nature Management. *Innlandsfiskeforvaltning 2010–2015. Oversikt over norsk innenlandsfiskeriforvaltning og naturforvaltningens strategier for 2010–2015*. Direktoratet for Naturforvaltning (DN)-rapport 6-2010 (Norwegian Directorate for Nature Management, 2010).

[22] Institute of Marine Research. Seafood data (Institute of Marine Research, 2021).

[23] Nerhus, I. et al. Iodine content of six fish species, Norwegian dairy products and hen’s egg. *Food Nutr. Res.* **62**10.29219/fnr.v62.1291 (2018). 10.29219/fnr.v62.1291 PMC5971469 29853825

[24] Reksten, A. M. et al. Sampling protocol for the determination of nutrients and contaminants in fish and other seafood—The EAF-Nansen Programme. *MethodsX* **7**, 24 (2020). 10.1016/j.mex.2020.101063 PMC7502570 32995313

[25] Norwegian Food Safety Authorities. (The Norwegian Food Composition Table, Norwegian Food Safety Authorities, Oslo, Norway, 2021).

[26] Dalane, J. Ø., Bergvatn, T. A. M., Kielland, E. & Carlsen, M. H. *Weight, Measure and Portion Sizes for Foods* (The Norwegian Food Safety Authority, University of Oslo, Directorate of Health, Oslo, Norway, 2015) (in Norwegian).

[27] Norwegian Directorate of Health. *Dietary Recommendations* https://www.helsedirektoratet.no/faglige-rad/kostradene-og-naeringsstoffer (2016).

[28] Evang, K. in *Selskapet for Norges Vel* (Det Kgl. selskap for Norges vel, Oslo, Norway, 1939).

[29] Oliveira, H. et al. Semi-industrial development of nutritious and healthy seafood dishes from sustainable species. *Food Chem. Toxicol.* **155**, 112431 (2021). 34293428 10.1016/j.fct.2021.112431

[30] Andersen, R. et al. Dietary effects of introducing school meals based on the New Nordic Diet—a randomised controlled trial in Danish children. The OPUS School Meal Study. *Br. J. Nutr.* **111**, 1967–1976 (2014). 24709026 10.1017/S0007114514000634

[31] Oyen, J. et al. Fatty fish intake and cognitive function: FINS-KIDS, a randomized controlled trial in preschool children. *BMC Med.* **16**, 10.1186/s12916-018-1020-z (2018). 10.1186/s12916-018-1020-z PMC5848440 29530020

[32] Vuholm, S. et al. Is high oily fish intake achievable and how does it affect nutrient status in 8–9-year-old children?: the FiSK Junior trial. *Eur. J. Nutr.* **59**, 1205–1218 (2020). 31073884 10.1007/s00394-019-01981-y

[33] EFSA Panel on Contaminants in the Food Chain. et al. Risk for animal and human health related to the presence of dioxins and dioxin‐like PCBs in feed and food. *EFSA J.* **16**, e05333 (2018). 32625737 10.2903/j.efsa.2018.5333 PMC7009407

[34] VKM et al. *Benefit and Risk Assessment of FIsh in the Norwegian diet*. Scientific Opinion of the Scientific Steering Committee of the Norwegian Scientific Committee for Food and Environment. VKM Report 17 (2022).

[35] Willett, W. et al. Food in the Anthropocene: the EAT-Lancet Commission on healthy diets from sustainable food systems. *The Lancet* **393**, 447–492 (2019). 10.1016/S0140-6736(18)31788-4 30660336

[36] Torell, M. et al. The role of seafood in sustainable and healthy diets. The EAT-Lancet Commission report through a blue lens. https://eatforum.org/content/uploads/2019/11/Seafood_Scoping_Report_EAT-Lancet.pdf (eatforum.org) (2019).

[37] Lombnæs, P., Bævre, O. A. & Vagstad, N. Norwegian agriculture: structure, research and policies. *Eur. J. Plant Sci. Biotechnol.* **5**, 1–4 (2011).

[38] Berg, H. S. F. & Nedreaas, K. H. *Estimering av utkast i norsk kystfiske med garn-2012–2018*. *Fisken og Havet* (Institute of Marine Research, 2021).

[39] Markhus, M. W. et al. Effects of two weekly servings of cod for 16 weeks in pregnancy on maternal iodine status and infant neurodevelopment: mommy’s food, a Randomized-Controlled Trial. *Thyroid* **31**, 288–298 (2021). 32746774 10.1089/thy.2020.0115 PMC7891220

[40] Christensen, J. J. et al. The Nordic Nutrition Recommendations 2022—principles and methodologies. *Food Nutr. Res.* **64**, 10.29219/fnr.v64.4402 (2020). 10.29219/fnr.v64.4402 PMC7307430 32612489

[41] Ziegler, F. et al. The carbon footprint of norwegian seafood products on the global seafood market. *J. Ind. Ecol.* **17**, 103–116 (2013).

[42] Meltzer, H. M. et al. Environmental sustainability perspectives of the Nordic diet. *Nutrients* **11**, 2248 (2019). 31540525 10.3390/nu11092248 PMC6769495

[43] Koehn, J. Z. et al. Fishing for health: do the world’s national policies for fisheries and aquaculture align with those for nutrition? *Fish. Fish.* **23**, 125–142 (2022).

[44] Ministry of Health and Care Services et al*. Policy—Nasjonal handlingsplan for bedre kosthold (2017–2021). Sunt kosthold, måltidsglede og god helse for alle!* Norwegian National Action Plan for a Healthier Diet (Ministry of Health and Care Services et al*.,* 2017).

[45] Hallenstvedt A. & Wulff I. *Norwegian Household Fishing in 2003,* Vol. 66 (Norwegian High School for Fisheries/University of Tromsø, Norway, 2004).

