# Supplementary material for: Small-scale fisheries contribution to food and nutrition security—a case study from Norway
Source: NPJ Ocean Sustain. 2022 Oct 5;1(1):5. doi: 10.1038/s44183-022-00005-3 (PMC9533999; doi:10.1038/s44183-022-00005-3)
Supplement: Supplementary file 4 — Supplementary Tables S1–S11 [file 44183_2022_5_MOESM4_ESM.docx]

Data was retrieved from the Seafood Data Base, <https://sjomatdata.hi.no/#search/> (25.02.2020). For iodine, values for cod, saith and haddock was retrieved from Nerhus et al, (2018). Detailed information about nutrient data are given in Table 1 – Table 9, for total mercury in Table 10 and dioxin and dl-PCBs in Table 11.

Table 1: Detailed information on analytical data for crude protein content in raw samples.

| Species | FAO code | Tissue analysed | Year analysed | N | g/100 grams | | | |
| --- | --- | --- | --- | --- | --- | --- | --- | --- |
|  |  |  |  |  | Mean | Median | Min | Max |
| Atlantic herring | HER | Fillet | 2006 | 10 | 19.9 | 17.8 | 17.5 | 18.3 |
| Atlantic mackerel | MAC | Fillet | 2006 | 10 | 16.9 | 16.9 | 15.7 | 17.6 |
| Atlantic cod | COD | Fillet | 2006 | 30 | 17.8 | 17.8 | 12.1 | 20.6 |
| Haddock | HAD | ND |  |  |  |  |  |  |
| Saithe | POK | Fillet | 2006 | 30 | 19.1 | 19.4 | 17.8 | 20.0 |
| Edible crab | CRE | Claw meet | 2011 | 11 | 16.3 | 15.2 | 8.20 | 24.3 |
| Red king crab | KCD | Claw meet | 2012 | 142 | 15.8 | 16.0 | 7.30 | 23.0 |
| Shrimp |  | Peeled | 2007 | 10 | 22.9 | 23.2 | 20.8 | 24.4 |

Atlantic cod: five additional samples were analysed 2007. Median values for these samples are 18.3 g/100 g.

Red king crab: Nine additional samples were analysed 2013. Median value for these samples were 16.0 g/100g.

ND: no data

Table 2: Detailed information on analytical data for total fat content in raw samples.

| Species | FAO code | Tissue analysed | Year analysed | N | g/100 grams | | | |
| --- | --- | --- | --- | --- | --- | --- | --- | --- |
|  |  |  |  |  | Mean | Median | Min | Max |
| Atlantic herring | HER | Fillet | 2017 | 100 | 11.8 | 12.5 | 3.30 | 21.0 |
| Atlantic mackerel | MAC | Fillet | 2019 | 160 | 18.6 | 18.0 | 2.20 | 37.0 |
| Atlantic cod | COD | Fillet | 2018 | 30 | 0.69 | 0.85 | 0.23 | 1.20 |
| Haddock | HAD | ND | 2014 | 31 | 0.91 | 0.93 | 0.18 | 1.30 |
| Saithe | POK | Fillet | 2006 | 30 | 1.43 | 1.40 | 1.00 | 2.10 |
| Edible crab | CRE | Claw meet | 2011 | 11 | 0.96 | 0.97 | 0.40 | 1.70 |
| Red king crab | KCD | Claw meet | 2012 | 141 | 1.43 | 1.50 | 0.67 | 2.10 |
| Shrimp |  | Peeled | 2019 | 7 | 2.39 | 2.50 | 2.00 | 2.70 |

ND: no data

Table 3: Detailed information on data for vitamin A_1_ content in raw samples.

| Species | FAO code | Tissue analysed | Year analysed | N | mg/100 grams | | | |
| --- | --- | --- | --- | --- | --- | --- | --- | --- |
|  |  |  |  |  | Mean | Median | Min | Max |
| Atlantic herring | HER | Fillet | 2006 | 10 | 0.036 | 0.038 | 0.011 | 0.054 |
| Atlantic mackerel | MAC | Fillet | 2006 | 10 | 0.012 | 0.010 | <LoQ | 0.020 |
| Atlantic cod | COD | Fillet | 2006 | 30 | 0.013 | 0.0051 | <LoQ | 0.064 |
| Haddock | HAD | Fillet | ND |  |  |  |  |  |
| Saithe | POK | Fillet | 2006 | 30 | 0.021 | 0.018 | <LoQ | 0.083 |
| Edible crab | CRE | Claw meet | ND |  |  |  |  |  |
| Red king crab | KCD | Claw meet | 2012 | 69 | <LoQ | <LoQ | <LoQ | <LoQ |
| Shrimp |  | Peeled | 2007 | 11 | <LoQ | <LoQ | <LoQ | 0.004 |

Atlantic cod: Two additional samples were analysed 2007. Median value for these samples were <LoQ.

ND: no data

Table 4: Detailed information on data for vitamin B_12_ content in raw samples.

| Species | FAO code | Tissue analysed | Year analysed | N | µg/100 grams | | | |
| --- | --- | --- | --- | --- | --- | --- | --- | --- |
|  |  |  |  |  | Mean | Median | Min | Max |
| Atlantic herring | HER | Fillet | 2012 | 96 | 12.0 | 12.0 | 7.60 | 20.0 |
| Atlantic mackerel | MAC | Fillet | 2012 | 71 | 9.61 | 9.90 | 6.40 | 14.0 |
| Atlantic cod | COD | Fillet | 2006 | 30 | 1.41 | 1.01 | 0.50 | 2.45 |
| Haddock | HAD | ND |  |  |  |  |  |  |
| Saithe | POK | Fillet | 2006 | 30 | 3.28 | 3.44 | 2.24 | 4.16 |
| Edible crab | CRE | Claw meet | 2011 | 11 | 3.96 | 4.60 | 0.26 | 7.90 |
| Red king crab | KCD | Claw meet | 2012 | 140 | 5.86 | 5.65 | 1.60 | 10.0 |
| Shrimp |  | Peeled | 2007 | 11 | 3.39 | 3.26 | 1.80 | 5.96 |

Atlantic herring: 100 additional samples were analysed 2011, 20 in 2010 and 10 in 2006. Median value for these samples were 8.45 µg/100 grams (2011), 12.5 µg/100 grams (2010) and 6.15 µg/100 grams (2006).

Atlantic mackerel: 274 additional samples were analysed 2011, 10 in 2010 and 10 in 2006. Median value for these samples were 9.20 µg/100 grams (2011), 9.93 µg/100 grams (2010) and 9.23 µg/100 grams (2006).

Atlantic cod: 10 additional samples were analysed 2010 and 3 in 2007. Median value for these samples were 1.13 µg/100 grams (2010) and 1.92 µg/100 grams (2007).

Saithe: 10 additional samples were analysed 2010. Median value for these samples were 3.96 µg/100 grams.

Edible crab: Nine additional samples were analysed 2013. Median value for these samples were 4.50 µg/100 grams.

ND: no data

Table 5: Detailed information on data for vitamin D_3_ contentin raw samples.

| Species | FAO code | Tissue analysed | Year analysed | N | mg/100 grams | | | |
| --- | --- | --- | --- | --- | --- | --- | --- | --- |
|  |  |  |  |  | Mean | Median | Min | Max |
| Atlantic herring | HER | Fillet | 2012 | 96 | 0.030 | 0.028 | 0.007 | 0.080 |
| Atlantic mackerel | MAC | Fillet | 2011 | 274 | 0.0040 | 0.004 | 0.001 | 0.009 |
| Atlantic cod | COD | Fillet | 2006 | 30 | <LoQ | <LoQ | <LoQ | 0.005 |
| Haddock | HAD | Fillet | ND |  |  |  |  |  |
| Saithe | POK | Fillet | 2006 | 30 | 0.0014 | <LoQ | <LoQ | 0.005 |
| Edible crab | CRE | Claw meet | 2011 | 11 | <LoQ | <LoQ | <LoQ | <LoQ |
| Red king crab | KCD | Claw meet | 2012 | 69 | <LoQ | <LoQ | <LoQ | <LoQ |
| Shrimp |  | Peeled | 2007 | 11 | <LoQ | <LoQ | <LoQ | <LoQ |

Atlantic herring: 100 additional samples were analysed 2011, 20 in 2010, 90 in 2007 and 20 in 2006. Median value for these samples were 0.020 mg/100 grams (2011), 0.0080 mg/100 grams (2010), 0.014 mg/100 grams (2007) and 0.0069 mg/100 g (2006).

Atlantic mackerel: 71 additional samples were analysed 2012, 10 in 2010, 20 in 2008, 79 in 2007 and 10 in 2006. Median value for these samples were 0.004 mg/100 grams (2012), 0.003 mg/100 grams (2010), 0.0068 mg/100 g (2008), 0.0032 mg/100 g (2007 and 0.0040 mg/100 grams (2006).

Atlantic cod: 10 additional samples were analysed 2010 and 2 in 2007. All median values were <LoQ.

Saithe: 10 additional samples were analysed 2010. Median value for these samples were <LoQ.

ND: no data

Table 6: Detailed information on data for iron content in raw samples.

| Species | FAO code | Tissue analysed | Year analysed | N | mg/100 grams | | | |
| --- | --- | --- | --- | --- | --- | --- | --- | --- |
|  |  |  |  |  | Mean | Median | Min | Max |
| Atlantic herring | HER | Fillet | 2017 | 125 | 1.04 | 1.00 | 0.61 | 3.90 |
| Atlantic mackerel | MAC | Fillet | 2017 | 72 | 0.87 | 0.84 | 0.53 | 1.30 |
| Atlantic cod | COD | Fillet | 2017 | 175 | 0.12 | 0.11 | 0.067 | 0.25 |
| Haddock | HAD | Fillet | 2014 | 167 | 0.13 | 0.10 | 0.060 | 2.20 |
| Saithe | POK | Fillet | 2017 | 71 | 0.37 | 0.36 | 0.22 | 1.20 |
| Edible crab | CRE | Claw meet | 2015 | 20 | 0.41 | 0.35 | 0.20 | 0.90 |
| Red king crab | KCD | Claw meet | 2012 | 327 | 0.40 | 0.40 | 0.10 | 0.90 |
| Shrimp |  | Peeled | 2018 | 6 | 0.84 | 0.49 | 0.34 | 2.60 |

Atlantic herring: 125 additional samples were analysed 2014, 50 in 2011, 484 in 2010, 500 in 2009, 699 in 2007 and 120 in 2006. Median value for these samples were 1.00 mg/100 grams (2014), 1.43 mg/100 grams (2011), 1.10 mg/100 grams (2010), 1.00 mg/100 g (2009), 1.20 (2007) and 1.05 mg/100 g (2006).

Atlantic mackerel: 150 additional samples were analysed 2016, 50 in 2015, 25 in 2014, 100 in 2013, 50 in 2012, 99 in 2009, 396 in 2008, 399 in 2007 and 25 in 2006. Median value for these samples were 0.91 mg/100 grams (2016), 1.15 mg/100 grams (2015), 1.00 mg/100 g (2014), 0.90 mg/100 g (2013), 1.35 mg/100 g (2012), 1.50 mg/100 g (2009), 0.91 mg/100 g (2008), 0.92 mg/100 g (2007) and 0.84 mg/100 grams (2006).

Atlantic cod: 222 additional samples were analysed 2016, 198 in 2015, 209 in 2014, 124 in 2013, 167 in 2012, 264 in 2011, 1164 in 2010, 681 in 2009, 100 in 2008, 99 in 2007 and 50 in 2006. Median value for these samples were 0.11 mg/100 grams (2016), 0.10 mg/100 grams (2015), 0.10 mg/100 g (2014), 0.20 mg/100 g (2013), 0.16 mg/100 g (2012), 0.19 mg/100 grams (2011) 0.15 (2010), 0.18 mg/100 g (2009), 0.16 mg/100 g (2008), 0.16 mg/100 g (2007) and 0.20 mg/100 grams (2006).

Haddock: 18 additonal samples were analysed 2015 and 28 in 2013. Median value was 0.10 mg/100 (2015) and 0.20 mg/100 g (2013).

Saith: 150 additional samples were analysed 2016, 117 in 2015, 100 in 2014, 222 in 2013, 540 in 2012, 347 in 2011, 586 in 2010 and 74 in 2006. Median value for these samples were 0.35 mg/100 grams (2016), 0.30 mg/100 grams (2015), 0.30 mg/100 g (2014), 0.30 mg/100 g (2013), 0.36 mg/100 g (2012), 0.41 mg/100 g (2011), 0.43 mg/100 g (2010) and 0.45 mg/100 grams (2006).

Edibel crab: 37 additional samples were analysed 2014 (0.30 mg/100 g) and 1 in 2011 (2.26 mg/100 g).

Red king crab: Nine additional sample was analysed 2013 with median concentration of 0.20 mg/100 g.

Shrimps: Seven additional samples were analysed 2017, 6 in 2016, 8 in 2015 and 2014, 5 in 2013, 7 in 2012, 3 in 2011 2010 and 2009, and 14 in 2007. Median value for these samples were 0.33 mg/100 grams (2017), 0.81 mg/100 grams (2016), 0.40 mg/100 g (2015), 0.50 mg/100 g (2014), 0.60 mg/100 g (2013), 1.00 mg/100 g (2012), 1.08 mg/100 g (2011), 1.10 mg/100 g (2010), 1.70 mg/100 g (2009), 0.44 mg/100 g (2008) and 0.77 mg/100 grams (2007).

ND: no data

Table 7: Detailed information on data for iodine content in raw samples. For Atlantic cod, haddock and saith, data from Nerhus et al, (2018) are used.

| Species | FAO code | Tissue analysed | Year analysed | N | mg/100 grams | | | |
| --- | --- | --- | --- | --- | --- | --- | --- | --- |
|  |  |  |  |  | Mean | Median | Min | Max |
| Atlantic herring | HER | Fillet | 2012 | 96 | 0.024 | 0.017 | 0.0087 | 0.25 |
| Atlantic mackerel | MAC | Fillet | 2012 | 71 | 0.020 | 0.019 | 0.0097 | 0.045 |
| Atlantic cod | COD | Fillet | 2014-2015 | 121 | 0.190 |  | 0.022 | 0.720 |
| Haddock | HAD | Fillet | 2014 | 65 | 0.400 |  | 0.035 | 2.200 |
| Saithe | POK | Fillet | 2013-2015 | 61 | 0.790 |  | 0.048 | 3.000 |
| Edible crab | CRE | Claw meet | 2011 | 11 | 0.099 | 0.11 | 0.029 | 0.18 |
| Red king crab | KCD | Claw meet | 2012 | 142 | 0.067 | 0.058 | 0.016 | 0.21 |
| Shrimp |  | Peeled | 2007 | 14 | 0.031 | 0.031 | 0.019 | 0.049 |

Atlantic herring: 100 additional samples were analysed 2011 and 20 in 2010. Median value for these samples were 0.016 mg/100 grams (2011) and 0.0056 mg/100 g (2010).

Atlantic mackerel: 274 additional samples were analysed 2011 and 10 in 2010. Median value for these samples were 0.021 mg/100 grams (2011) and 0.016 mg/100 grams (2010).

Atlantic cod: Ten samples were analysed in 2010 and 2007. Median values for these samples were 0.068 mg/100 g (2010) and 0.092 mg/100 g (2007).

Saith: 10 samples were analysed in 2010. Median value for these samples were 0.26 mg/100 grams.

Red king crab: Nine additional sample was analysed 2013 with median concentration of 0.26 mg/100 g.

Table 8: Detailed information on data for zinc content in raw samples.

| Species | FAO code | Tissue analysed | Year analysed | N | mg/100 grams | | | |
| --- | --- | --- | --- | --- | --- | --- | --- | --- |
|  |  |  |  |  | Mean | Median | Min | Max |
| Atlantic herring | HER | Fillet | 2017 | 125 | 0.68 | 0.66 | 0.44 | 1.10 |
| Atlantic mackerel | MAC | Fillet | 2017 | 72 | 0.59 | 0.57 | 0.36 | 1.10 |
| Atlantic cod | COD | Fillet | 2017 | 175 | 0.38 | 0.37 | 0.28 | 0.64 |
| Haddock | HAD | Fillet | 2014 | 167 | 0.31 | 0.30 | 0.20 | 0.40 |
| Saithe | POK | Fillet | 2017 | 71 | 0.46 | 0.45 | 0.31 | 0.65 |
| Edible crab | CRE | Claw meet | 2014 | 37 | 8.06 | 7.60 | 3.60 | 33.0 |
| Red king crab | KCD | Claw meet | 2012 | 327 | 4.00 | 3.80 | <LoQ | 6.50 |
| Shrimp |  | Peeled | 2018 | 6 | 1.47 | 1.40 | 1.40 | 1.70 |

Atlantic herring: 125 additional samples were analysed 2014, 50 in 2011, 484 in 2010, 500 in 2009, 699 in 2007 and 120 in 2006. Median value for these samples were 0.70 mg/100 grams (2014), 0.83 mg/100 grams (2011), 0.67 mg/100 grams (2010), 0.59 mg/100 g (2009), 0.67 (2007) and 0.40 mg/100 g (2006).

Atlantic mackerel: 150 additional samples were analysed 2016, 50 in 2015, 25 in 2014, 100 in 2013, 50 in 2012, 10 in 2010, 99 in 2009, 396 in 2008, 399 in 2007 and 25 in 2006. Median value for these samples were 0.62 mg/100 grams (2016), 0.60 mg/100 grams (2015), 0.50 mg/100 g (2014), 0.60 mg/100 g (2013), 0.90 mg/100 g (2012), 0.44 (2010), 0.83 mg/100 g (2009), 0.63 mg/100 g (2008), 0.62 mg/100 g (2007) and 0.58 mg/100 grams (2006).

Atlantic cod: 222 additional samples were analysed 2016, 198 in 2015, 209 in 2014, 124 in 2013, 167 in 2012, 264 in 2011, 1164 in 2010, 681 in 2009, 100 in 2008, 99 in 2007 and 50 in 2006. Median value for these samples were 0.36 mg/100 grams (2016), 0.40 mg/100 grams (2015), 0.40 mg/100 g (2014), 0.30 mg/100 g (2013), 0.40 mg/100 g (2012), 0.34 mg/100 grams (2011) 0.36 (2010), 0.38 mg/100 g (2009), 0.36 mg/100 g (2008), 0.35 mg/100 g (2007) and 0.31 mg/100 grams (2006).

Haddock: 18 samples were analysed in 2015 and 28 in 2013. Median value for these samples were 0.30 mg/100 grams at both time points.

Saith: 150 additional samples were analysed 2016, 117 in 2015, 100 in 2014, 222 in 2013, 540 in 2012, 347 in 2011, 586 in 2010 and 74 in 2006. Median value for these samples were 0.43 mg/100 grams (2016), 0.40 mg/100 grams (2015), 0.40 mg/100 g (2014), 0.40 mg/100 g (2013), 0.40 mg/100 g (2012), 0.43 mg/100 grams (2011) 0.52 (2010) and 0.42 mg/100 grams (2006).

Edible crab: 20 additional samples were analysed 2015, 1 in 20100, 19 in 2010, 2 in 2009, 4 in 2008 and 7 in 2007. Median values for these samples were 7.95 mg/100 g (2015), 9.31 mg/100 g (2011), 7.40 mg/100 g (2010), 6.70 mg/100 g (2009), 8.90 mg/100 g (2008) and 6.70 mg/100 g (2007).

Red king crab: Nine additional sample was analysed 2013 with median concentration of 2.30 mg/100 g.

Shrimps: Seven additional samples were analysed 2017, 6 in 2016, 8 in 2015 and 2014, 5 in 2013, 7 in 2012, 3 in 2011, 2010, 2009 and 2008 and 14 in 2007. Median value for these samples were 1.30 mg/100 grams (2017), 1.40 mg/100 grams (2016), 1.50 mg/100 g (2015), 1.25 mg/100 g (2014), 1.30 mg/100 g (2013), 1.30 mg/100 g (2012), 1.26 mg/100 g (2011), 1.33 mg/100 g (2010), 1.50 mg/100 g (2009), 1.20 mg/100 g (2008) and 1.25 mg/100 grams (2007).

Table 9: Detailed information on data for docosahexaenoic acid (DHA) content in fresh samples.

| Species | FAO code | Tissue analysed | Year analysed | N | mg/100 grams | | | |
| --- | --- | --- | --- | --- | --- | --- | --- | --- |
|  |  |  |  |  | Mean | Median | Min | Max |
| Atlantic herring | HER | Fillet | 2012 | 96 | 712 | 688 | 252 | 1700 |
| Atlantic mackerel | MAC | Fillet | 2012 | 71 | 2180 | 2030 | 581 | 4580 |
| Atlantic cod | COD | Fillet | 2006 | 30 | 211 | 211 | 123 | 307 |
| Haddock | HAD | Fillet | ND |  |  |  |  |  |
| Saithe | POK | Fillet | 2006 | 30 | 308 | 307 | 194 | 464 |
| Edible crab | CRE | Claw meet | 2011 | 10 | 48.1 | 51.5 | 24.0 | 68.0 |
| Red king crab | KCD | Claw meet | 2012 | 142 | 108 | 108 | 38.0 | 222 |
| Shrimp |  | Peeled | 2007 | 12 | 174 | 167 | 148 | 229 |

Atlantic herring: 100 additional samples were analysed 2011, 19 in 2010 and 10 in 2006. Median value for these samples were 1560 mg/100 grams (2011), 1190 mg/100 grams (2010) and 1230 mg/100 g (2006).

Atlantic mackerel: 274 additional samples were analysed 2011, 10 in 2010 and 10 in 2006. Median value for these samples were 3180 mg/100 grams (2011), 2810 mg/100 grams (2010) and 3770 mg/100 grams (2006).

Atlantic cod: 10 additional samples were analysed 2010 and 2007. Median value for these samples were 170 mg/100 grams (2007) and 186 mg/100 grams (2006).

Saithe: 10 samples were analysed in 2010. Median value for these samples were 350 mg/100 grams.

Red king crab: Nine additional sample was analysed 2013 with median concentration of 87.0 mg/100 g.

ND: no data

Table 10: Detailed information on data for mercury content in fresh samples. Threshold value = 0.50 mg/kg.

| Species | FAO code | Tissue analysed | Year analysed | N | mg/kg | | | |
| --- | --- | --- | --- | --- | --- | --- | --- | --- |
|  |  |  |  |  | Mean | Median | Min | Max |
| Atlantic herring | HER | Fillet | 2017 | 125 | 0.053 | 0.048 | <0.0030 | 0.12 |
| Atlantic mackerel | MAC | Fillet | 2019 | 160 | 0.048 | 0.039 | 0.018 | 0.19 |
| Atlantic cod | COD | Fillet | 2019 | 97 | 0.010 | 0.096 | 0.010 | 0.54 |
| Haddock | HAD | Fillet | 2014 | 168 | 0.014 | 0.045 | 0.014 | 0.41 |
| Saithe | POK | Fillet | 2016 | 150 | 0.059 | 0.051 | 0.016 | 0.22 |
| Edible crab | CRE | Claw meet | 2014 | 37 | 0.11 | 0.11 | 0.026 | 0.41 |
| Red king crab | KCD | Claw meet | 2012 | 327 | 0.0020 | 0.035 | 0.0020 | 0.14 |
| Shrimp |  | Peeled | 2019 | 8 | 0.084 | 0.066 | 0.038 | 0.16 |

Table 11: Detailed information on data for dioxins and dl-PCBs content in fresh samples. Threshold value is 6.50 nanogram TEQ/kg.

| Species | FAO code | Tissue analysed | Year analysed | N | Nanogram TEQ/kg | | | |
| --- | --- | --- | --- | --- | --- | --- | --- | --- |
|  |  |  |  |  | Mean | Median | Min | Max |
| Atlantic herring | HER | Fillet | 2017 | 50 | 0.89 | 0.86 | 0.29 | 1.48 |
| Atlantic mackerel | MAC | Fillet | 2018 | 184 | 0.78 | 0.53 | 0.14 | 8.11 |
| Atlantic cod | COD | Fillet | 2019 | 15 | 0.022 | 0.037 | 0.022 | 0.075 |
| Haddock | HAD | Fillet | 2014 | 31 | 0.065 | 0.059 | 0.039 | 0.12 |
| Saithe | POK | Fillet | 2006 | 41 | 0.11 | 0.097 | 0.054 | 0.21 |
| Edible crab | CRE | Claw meet | 2006 | 2 | 0.22 |  | 0.22 | 0.32 |
| Red king crab | KCD | Claw meet | 2012 | 49 | 0.13 | 0.13 | 0.055 | 0.38 |
| Shrimp |  | Peeled | 2019 | 8 | 0.20 | 0.19 | 0.079 | 0.38 |
